# The role of acroblast formation during *Drosophila* spermatogenesis

**DOI:** 10.1242/bio.018275

**Published:** 2016-08-01

**Authors:** Karolina Fári, Sándor Takács, Dániel Ungár, Rita Sinka

**Affiliations:** 1 Department of Genetics, University of Szeged, Szeged 6726, Hungary; 2 Department of Biology, University of York, York YO10 5DD, UK

**Keywords:** Acrosome, Acroblast, Golgi, *Drosophila*, Spermatogenesis

## Abstract

Protein recycling is important for maintaining homeostasis of the Golgi and its cisternae. The Vps54 (Scat) protein, a subunit of the GARP tethering complex, is a central factor in retrograde transport to the *trans*-Golgi. We found the *scat^1^* mutant to be male sterile in *Drosophila* with individualization problems occurring during spermatogenesis. Another typically observed phenotype was the abnormal nuclear structure in elongated mutant cysts. When examining the structure and function of the Golgi, a failure in acrosome formation and endosome-Golgi vesicular transport were found in the *scat^1^* mutant. This acrosome formation defect was due to a fault in the *trans*-Golgi side of the acroblast ribbon. When testing a mutation in a second retrograde transport protein, Fws, a subunit of the conserved oligomeric Golgi (COG) tethering complex, the acroblast structure, was again disrupted. *fws^P^* caused a similar, albeit milder, acrosome and sperm individualization phenotype as the *scat^1^* mutant. In the case of *fws^P^* the *cis* side of the acroblast ribbon was dispersed, in-line with the intra-Golgi retrograde function of COG. Our results highlight the importance of an intact acroblast for acrosome formation, nuclear elongation and therefore sperm maturation. Moreover, these results suggest the importance of retrograde tethering complexes in the formation of a functional Golgi ribbon.

## Abstract (precis)

**Summary:** This study demonstrates that retrograde tethering complexes are necessary to form a functional acroblast, which is essential for normal nuclear elongation and acrosome formation during *Drosophila* spermatogenesis.

## INTRODUCTION

In most mammalian cell types the Golgi apparatus appears as a ribbon formed from interconnected stacks of cisternae, however, this arrangement is not universal. For example, in gastric parietal cells the Golgi appears in the form of mini-stacks dispersed throughout the cytoplasm (Gunn et al., 2011). This scattered arrangement is common in most cell types of the fruit fly *Drosophila* *melanogaster* (Kondylis and Rabouille, 2009). Several possible roles have been proposed for the assembly of Golgi stacks into a ribbon. These include an increase in efficiency and uniformity of glycosylation (Puthenveedu et al., 2006), a necessity of the ribbon for the secretion of large cargoes (Lavieu et al., 2014), and importantly, a role in polarized secretion (Horton et al., 2005). Generation of the Golgi ribbon requires microtubule-mediated transport of stacks, or the vesicles that form them, into the vicinity of the microtubule organizing centre (Wehland et al., 1983), followed by tethering and fusion into a ribbon (Marra et al., 2007). Specialized cell types in non-vertebrates can also present an assemblage of Golgi stacks in a perinuclear location. For example, the Golgi apparatus of developing *Drosophila* spermatids is in a peri-nuclear location just prior to and during the nuclear elongation phase of spermatogenesis (Kondylis and Rabouille, 2009). This specialized Golgi assemblage, known as the acroblast, is likely to be needed to organize the secretory pathway in this highly polarized cell type of the fruit fly.

While it is clear that anterograde transport to the Golgi is essential for generation of a polarized assembly of Golgi stacks, the role of retrograde transport in this process is less well understood. Retrograde transport within and to the Golgi is coordinated by two multisubunit tethering complexes, the Golgi associated retrograde protein (GARP) (Bonifacino and Hierro, 2011) and the conserved oligomeric Golgi (COG) (Miller and Ungar, 2012) complexes. GARP is a four subunit complex of the complexes associated with tethering containing helical rods (CATCHR) family (Hughson and Reinisch, 2010), composed of the Vps51, Vps52, Vps53 and Vps54 proteins (Conibear and Stevens, 2000). Its primary role in membrane trafficking is to direct retrograde carriers to the *trans*-Golgi network (TGN) (Conibear and Stevens, 2000), such as vesicles that recycle the mannose-6-phosphate receptor (M6PR) (Perez-Victoria et al., 2008), or those carrying the SNARE protein Snc1 (Quenneville et al., 2006) from endosomes to the TGN. Lack of Vps54 in mice causes the *wobbler* phenotype, which manifests in progressive neurodegeneration and male sterility (Schmitt-John et al., 2005). In *Drosophila* the GARP complex has been shown to require the Arl5 GTPase for correct localization, loss of which results in defective recycling of Lerp, the fly homolog of M6PR (Rosa-Ferreira et al., 2015). While COG is also involved in endosome-to-Golgi transport (Whyte and Munro, 2001), its main function is the intra-Golgi retrograde trafficking of resident Golgi proteins (Oka et al., 2004). The eight COG subunits can be grouped into two lobes, with subunits Cog1-4 forming lobe A, and Cog5-8 lobe B (Ungar et al., 2002). While loss of lobe A function causes defects in the recycling of early Golgi residents, lobe B is mainly involved in late Golgi homeostasis (Oka et al., 2004; Willett et al., 2013; Wu et al., 2004). Consequently, lobe A is essential for development of an organism as its loss is lethal in yeast (Whyte and Munro, 2001) and during early development in *Drosophila* (Schnorrer et al., 2010). In contrast, lobe B loss causes much milder phenotypes, for example loss of Cog5 in a human patient was shown to lead to relatively mild psychomotor retardation (Paesold-Burda et al., 2009), while loss-of-function alleles of its fly homolog, fws, cause male sterility due to incomplete cytokinesis during spermatogenesis (Farkas et al., 2003). Interestingly, COG interacts with the golgin TMF (Miller et al., 2013), which is a critical factor for vesicular transport during late stages of mouse spermatogenesis (Lerer-Goldshtein et al., 2010).

**Fig. 1.**
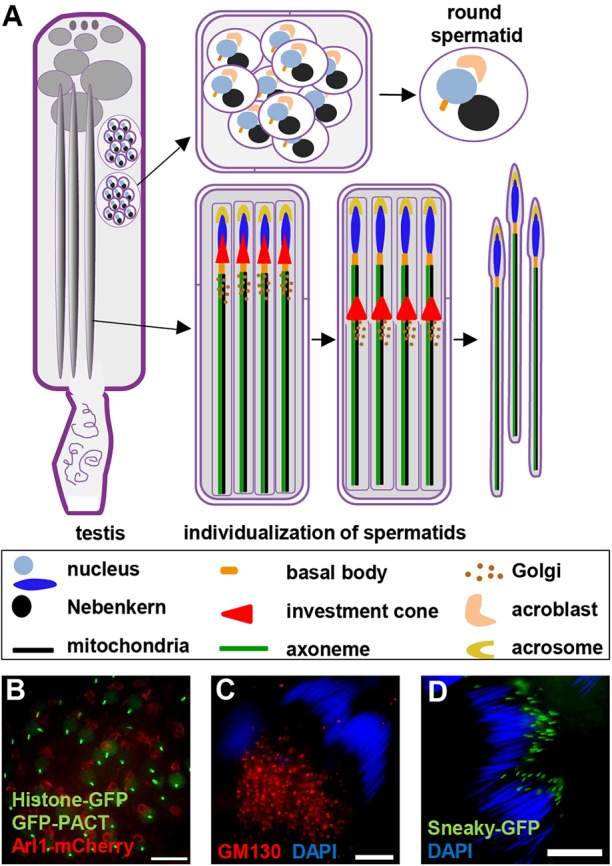
**Post-meiotic *Drosophila* spermatogenesis.** (A) Schematic representation of spermatogenesis highlighting round and individualizing spermatids. (B) Fluorescent images of spermatids co-expressing Arl1-mCherry (acroblast, red), GFP-PACT (basal body, green) and Histone-GFP (nucleus, green). (C) Confocal images of elongated cysts with antibody staining of the *cis*-Golgi marker GM130 (red) localizing at the basal end of the cysts. (D) Fluorescent images of Snky-GFP (green) in elongated 64-cell cysts marking the formed acrosome. Nuclei labelled with DAPI (blue) in C,D. Scale bars: 10 µm in B-D.

During *Drosophila* spermatogenesis, following meiotic division, the 64 spermatids undergo a dramatic differentiation program that leads to formation of the highly elongated flagellated mature sperm (Fig. 1A). This process starts with rearrangement and fusion of mitochondria to form the Nebenkern from two mitochondrial derivatives (Fig. 1A) (Tokuyasu et al., 1972). At the same time the basal body is embedded into the nuclear envelope to polarize the nucleus (Vogt et al., 2006). The Golgi apparatus, which is normally a collection of scattered stacks throughout the cytosol, is then recruited to the nucleus at the opposing side to the basal body (Fig. 1A,B) (Fuller, 1993). This polarization event is thought to be essential for subsequent nuclear elongation. The change in nuclear shape is coincident with a major reorganization of chromatin, which manifests in the replacement of histones with protamines. This histone to protamine switch is critical for the proper elongation of the nuclei (Raja and Renkawitz-Pohl, 2005). During the later stages of nuclear elongation the specialized Golgi structure, the acroblast, is converted into the acrosome and the actin-based investment cones are formed (Fig. 1A). These investment cones are also involved in the individualization of the mature sperm when an enormous amount of new membrane is used for elongation, which concludes spermatogenesis (Fabian and Brill, 2012).

The acroblast (described above) contains all the markers of a typical Golgi apparatus, such as the glycosylation enzyme mannosidase II (Farkas et al., 2003); the golgins Golgin245, GM130 (Hirst and Carmichael, 2011) and Lava lamp (Farkas et al., 2003); the COPI vesicle coat (Kitazawa et al., 2012) and the COG complex (Farkas et al., 2003). In addition, the lysosomal protein Lamp1, and the acrosomal protein Sneaky also localize to the acroblast (Wilson et al., 2006) (Fig. S1G,H). Yet, the acroblast is unusual in *Drosophila,* as it forms a ribbon as opposed to the scattered stacks typical for Golgi architecture in other fruit fly cells (Kondylis and Rabouille, 2009). The molecular determinants of acroblast formation and its breakdown upon acrosome formation are not very well understood, but the Golgi architecture leading to acrosome formation has been recently documented (Yasuno et al., 2013). After meiosis the Golgi is organized around the nucleus and participates in the formation of the acroblast (Fig. 1A,B), once the nuclei elongate the acroblast disassembles and some of the Golgi components, such as Sneaky, together with lysosomal components generate the acrosome which maintains an apical positioning next to the nucleus (Fig. 1A,D). At the same time the remaining Golgi components migrate to the posterior side of the nucleus and appear as scattered stacks akin to somatic *Drosophila* cells (Fig. 1A,C). The known molecular players that have so far been associated with the formation of the acroblast, and its later breakdown, have all been found to affect meiotic division as well (Belloni et al., 2012; Farkas et al., 2003). It is therefore often difficult to tease out direct effects on Golgi architecture from secondary effects due to delays in spermatogenesis and associated defects in polarization. Such factors include microtubules (Yasuno et al., 2013), the phosphatidylinositol transfer protein Giotto (Giansanti et al., 2006), the small GTPase Rab11 (Giansanti et al., 2007), the TRAPP II complex (Robinett et al., 2009), as well as the Cog5 and Cog7 subunits of COG (Belloni et al., 2012; Farkas et al., 2003).

Here we have analyzed two different male sterile P-element insertion mutations; one of the GARP subunit Vps54 (*scat*), the other of the Cog5 (*fws*) subunit of COG. These mutants have no defects in the meiotic phase of sperm development, but nuclear elongation and acrosome formation are both affected. Mutant spermatids of *scat^1^* and *fws^P^* do not individualize and therefore do not mature. We show that the main defect of these mutants is in the organization of the acroblast and the ensuing completion of the spermatogenic differentiation program. These results highlight an essential function of the GARP and COG mediated retrograde transport processes in the establishment of a polarized Golgi ribbon, which is important in nuclear elongation, individualization and acrosome formation during *Drosophila* spermatogenesis.

## RESULTS

### The *scat^1^* mutant has a male fertility defect

**Fig. 2.**
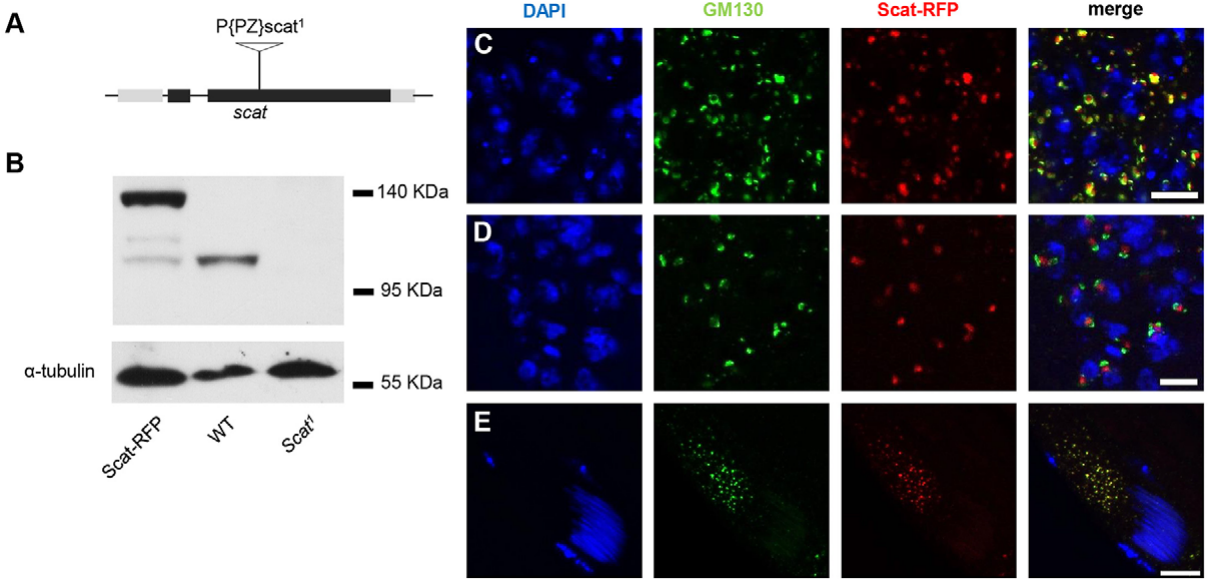
***Scat^1^* is a null mutant and Scat protein shows Golgi and acroblast localization in *Drosophila* testis.** (A) Schematic representation of the *scat* gene with the inserted P element in the third exon. (B) Immunoblots of testis lysates from WT, *scat^1^* mutant, and WT expressing a Scat-RFP transgene. α-Tubulin is used as a loading control. (C-E) Confocal images of Scat-RFP (red) expressing testes immunostained for dGM130 (green). (C) primary spermatocytes, (D) meiotic spermatids, (E) elongated spermatids. Nuclei labelled with DAPI (blue). Scale bars: 10 µm.

In order to probe the function of vesicle tethering complexes that act at the Golgi during spermatogenesis, we investigated the Vps54 subunit of the GARP complex, encoded by the *scat* gene. The *scat^1^* allele was identified as a male sterile mutant with scattered nuclei in a P element screen (Castrillon et al., 1993). The P element is incorporated in the third exon of the gene (Fig. 2A). Genetic characterization showed that homozygous *scat^1^* males were 100% sterile and their seminal vesicle was devoid of mature sperm (Fig. S1A,B); in contrast, all females were fertile. We tested the *scat^1^* allele in complementation analysis and found male sterility in a hemizygous combination with an overlapping *Df(2L)ED680* deficiency. The male sterility of *scat^1^* was completely reversed by precise excision of the P element. To verify the involvement of the *scat* gene a C-terminally RFP tagged scat transgene expressed from a *P{UASp}* vector was used to rescue the male sterile phenotype. Expression of the *P{UASp-Scat-RFP}* fusion protein using the germ line specific *Bam-Gal4* driver completely rescued male sterility (Fig. S1G-L). This proves that the P element insertion within *scat* is indeed responsible for the male sterility and the Scat-RFP fusion protein correctly incorporates into the GARP complex. A polyclonal antibody raised against Scat recognizes the protein at the predicted molecular weight of 105 kDa, as well as the Scat-RFP fusion protein in extracts from wild-type or Scat-RFP transgenic testes (Fig. 2B, first two lanes). In contrast, in homozygous *scat^1^* mutant testis extracts the protein was absent from the immunoblot, confirming that *scat^1^* is a null mutant (Fig. 2B, right lane).

### Scat is Golgi localized throughout spermatogenesis

**Fig. 3.**
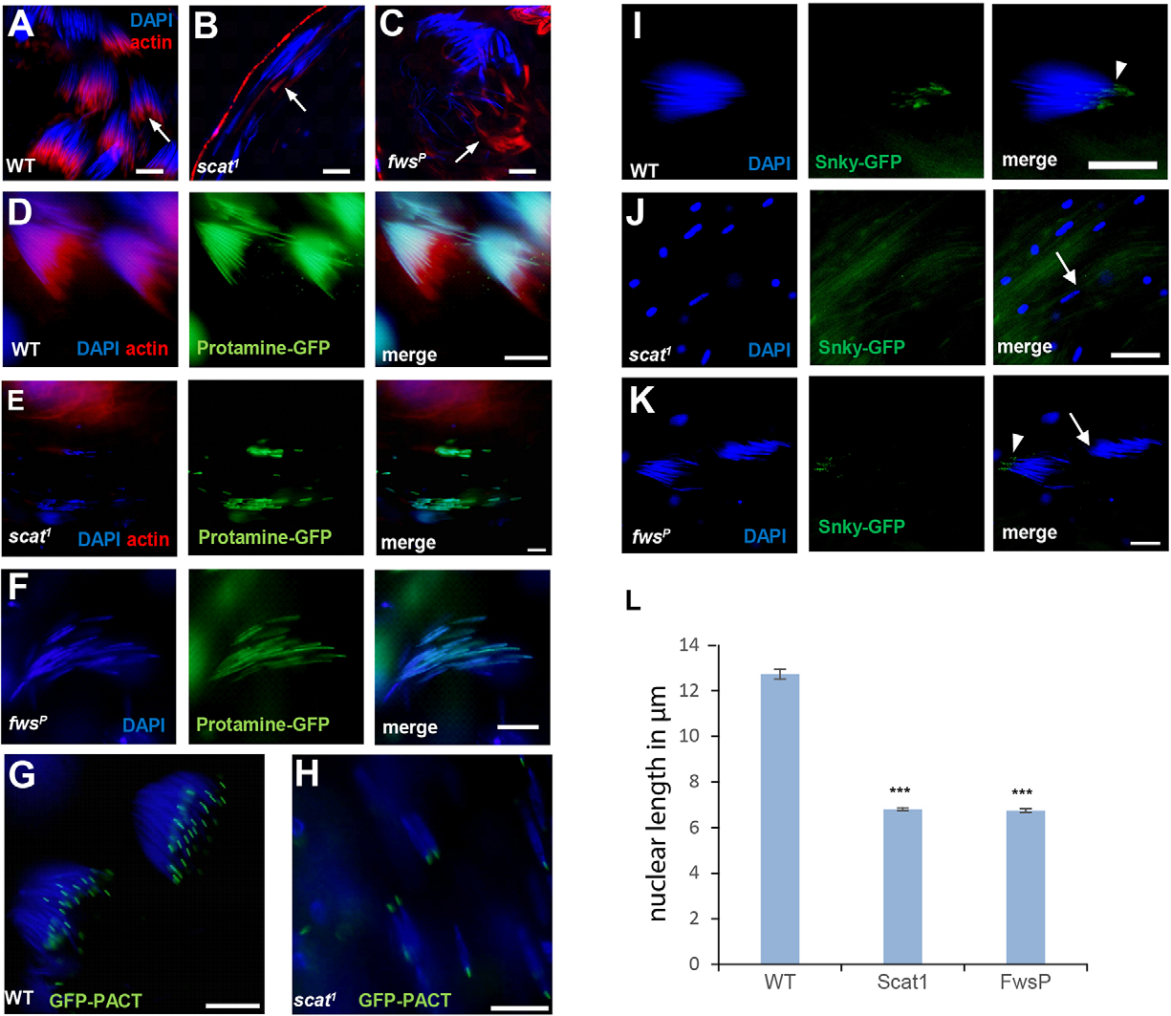
**The main defects are the failure to complete nuclear elongation followed by scattering of the nuclei and defective acrosome organization in *scat^1^* and *fws^P^* mutants.** (A-C) Investment cones (arrows) visualized by confocal microscopy using Phalloidin staining (red) and DAPI (blue) in WT, *scat^1^* and *fws^P^* spermatids containing elongated nuclei. (D-F) Protamine-GFP (green) expressed in elongating spermatids visualized in WT (D), *scat^1^* (E) and *fws^P^* (F) mutants using confocal microscopy. (G,H) Basal bodies visualized with GFP- PACT (green) in WT (G) and *scat^1^* mutant (H) cysts. (I-K) Fluorescence images of elongated spermatids visualized with Snky-GFP in WT (I), *scat^1^* (J) and *fws^P^* (K) mutants. Arrowheads label acrosomes in the WT and *fws^P^* mutant. Arrows label the lack of acrosome in *scat^1^* and *fws^P^* mutant. Nuclei labelled with DAPI (blue). Scale bars: 10 µm. (L) Measurement of nuclear length in elongated spermatids. *n*=100 in each genotype, ***P<0.001, one-way ANOVA, mean±s.e.m. Length is indicated in µm.

Mouse Vps54 was shown to localize to both endosomes and Golgi, and to incorporate into the fully developed acrosome (Berruti et al., 2010). This is in contrast with other Golgi trafficking proteins, such as Golgin95 or Golgin97, which localize to the developing acrosome only during the early steps of acrosomogenesis but do not label the testicular spermatozoa (Moreno et al., 2000). We therefore tested the subcellular localization of Scat-RFP during different stages of *Drosophila* spermatogenesis. Both in the early premeiotic and in the late postmeiotic stages Scat localized to the Golgi (Fig. 2C-E), as is typical for the GARP complex (Conibear et al., 2003). This was confirmed by co-staining with anti-dGM130, a protein known to be restricted to the *cis*-Golgi cisternae, just as its mammalian orthologue (Fig. 2C-E) (Sinka et al., 2008). We found that the Scat-RFP signal localized close but slightly displaced from dGM130 in all stages of spermatogenesis, suggesting that Scat is localized to the *trans* side of the Golgi (Fig. 2C-E). Early in the development process, in primary spermatocytes, the RFP stained Golgi is randomly distributed throughout the cytoplasm, similarly to the distribution found for GM130 and other medial/*trans* Golgi markers (Yasuno et al., 2013) (Fig. 2C). Interestingly after meiosis, during acroblast formation the RFP staining marking the *trans* side of the Golgi was always positioned in the proximity of the nuclei as opposed to the more distally positioned *cis*-Golgi side (Fig. 2D). During nuclear elongation the Golgi localized Sneaky and the lysosomal Lamp then localized to the acrosome (Fig. 3I; Fig. S2A), but Scat, like the *cis*-Golgi specific dGM130, and the *trans*-Golgi specific dGolgin245 did not. Rather, Scat localized with the rest of the Golgi markers in the scattered Golgi-stacks that moved to the basal side of the nucleus and were later removed in the cystic bulge with the majority of the cytosol (Fig. 2E).

### Nuclear elongation is disrupted in *scat^1^* males

All early steps of spermatogenesis, such as the maintenance of germ stem cells, the formation of primary spermatocytes, and meiotic divisions were normal in *scat^1^* testes. Nucleus to Nebenkern ratio was 1:1 in all round spermatids of the *scat^1^* mutant, suggesting normal cytokinesis (Fig. S1D,E). We therefore focussed on the post-meiotic stages of spermatogenesis to understand how the loss of Scat function perturbs spermatogenesis. Investigating the elongating spermatid nuclei their majority were found in late canoe stage and hardly any were observed as needle shaped in the *scat^1^* mutant cysts (Fig. 3A,B,D,E,G-J). The lack of needle shaped, fully elongated nuclei correlated with the appearance of scattered spermatid bundles in the *scat^1^* mutant post-meiotic cysts (Fig. 3A,B). Elongation and chromatin condensation occur parallel to each other. As in mammals, chromatin condensation is achieved by a histone to protamine switch during nuclear elongation in *Drosophila* (Raja and Renkawitz-Pohl, 2005). This switch is normal in the *scat^1^* mutant (Fig. 3D,E), suggesting that the observed nuclear elongation defect is independent of the chromatin condensation process. The scattering of nuclei could also be caused by defects in basal body formation (Texada et al., 2008). However, visualization of the basal body with GFP-PACT failed to reveal any abnormalities in elongated cysts of the *scat^1^* mutant (Fig. 3G,H).

Vps54 mutant mice that are male sterile are missing acrosomes (Paiardi et al., 2011). These are normally formed during the later stages of nuclear elongation, so we wondered whether acrosome formation was normal in *scat^1^* mutants. Two different acrosomal markers, Snky-GFP and Lamp1-GFP (Fabian and Brill, 2012; Wilson et al., 2006) both showed acrosomal localization at the tips of elongated nuclei in WT spermatids (Fig. 3I; Fig. S2A). Yet the GFP signal was diffuse without any recognizable acrosome staining in the same stage of *scat^1^* mutant spermatids (Fig. 3J; Fig. S2B).

Individualization starts with the formation of 64 actin-rich investment cones adjacent to the nuclei, which move together towards the distal end of the individualizing cyst (Fig. 3A) (Fabrizio et al., 1998). In the case of the *scat^1^* mutant we observed hardly any investment cones and the process of individualization did not start. Occasionally we could detect a very faint Phalloidin signal, which could be due to investment cone remnants or the investment cones in the process of degradation, but these were always scattered (Fig. 3B). Following failed individualization, the elongated cysts lost their integrity and the cells scattered and died.

Thus the earliest defect in spermatogenesis in *scat^1^* mutants is their failure to fully elongate the nuclei. While this is not accompanied with a defect in chromatin condensation, it does lead to a defect in individualization.

### Acrosome defects are the consequence of the abnormal acroblast formation

**Fig. 4.**
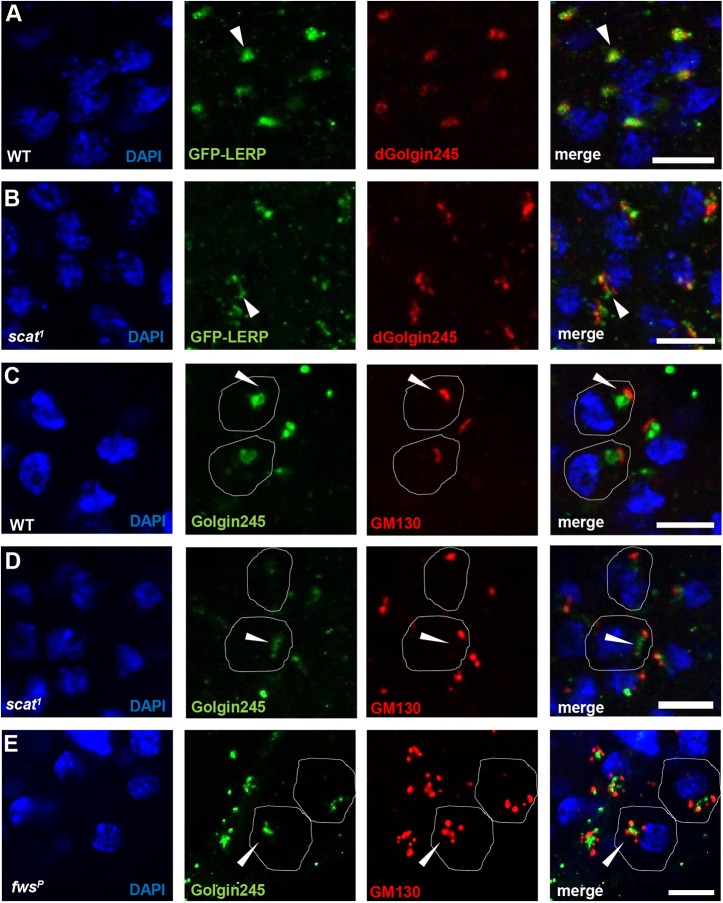
**Integrity of the Golgi ribbon is compromised at the acroblast stage in the *scat^1^* and *fws^P^* mutants.** (A,B) Confocal micrographs of round spermatids expressing GFP-Lerp (green) immunostained for dGolgin245 (red) in WT (A) and in *scat^1^* (B) mutant. (C-E) Confocal micrographs of WT (C), *scat^1^* (D) and *fws^P^* (E) round spermatids stained with the Golgi markers Golgin245 (green) and GM130 (red). Nuclei are marked with DAPI (blue). Scale bars: 10 µm. Arrowheads label acroblast.

The failures in nuclear elongation, acrosome formation and individualization all point to a defect following acroblast disassembly. This could be caused by an inherent defect of the acroblast itself in the mutants. The GARP complex is known to contribute to the recycling of M6PR between endosomes and the TGN (Perez-Victoria et al., 2008) in mammals, and therefore we wondered if this trafficking pathway was, for example, defective at the acroblast. A transgenic line was established with testis-specific expression of the *Drosophila* M6PR, *P{tv3-GFP-Lerp}*. This showed acroblast localization in WT spermatids (Fig. 4A), while it had a more dispersed localization in the *scat^1^* mutant (Fig. 4B). The *trans*-Golgi marker Golgin245 showed co-localization with GFP-Lerp in WT, but not in the *scat^1^* mutant postmeiotic round spermatids (Fig. 4A,B). Thus similar to effects in mammalian cells, a faulty GARP complex causes defective retrograde trafficking of M6PR from endosomes to the TGN. Such a defect in the endosome-Golgi trafficking route marked by the M6PR could affect acroblast integrity.

The distribution of *cis-* and *trans-*Golgi markers was therefore tested throughout spermatogenesis. In the early stage the *cis*- and *trans*-Golgi markers GM130 and Golgin245 are localized close to each other in both WT and *scat^1^* mutant spermatocytes (Fig. S2D,F). In WT post-meiotic spermatids these *cis*- and *trans*-Golgi markers appear closely apposed to each other in perinuclear localization, consistent with the perinuclear ribbon-like Golgi formed by the acroblast (Fig. 4C). However, in the *scat^1^* mutants of the same stage, localization of the *trans*-Golgi marker is much more diffuse (Fig. 4D). The failure of GM130 and Golgin245 to co-localize in the *scat^1^* mutants persists during nuclear elongation when the Golgi travels to the basal side of the nucleus (Fig. S2E,G). However, the high degree of cell death at this stage precludes far reaching conclusions to be drawn from this last stage. Overall these results suggest that a functional GARP complex is necessary for normal acroblast organization. Our data also imply that proper perinuclear organization of the acroblast is required for completion of spermatogenesis, including individualization, acrosome formation and the final stage of nuclear elongation.

### The intra-Golgi retrograde transport factor Fws is also necessary for acroblast integrity and completion of the late stages of spermatogenesis

Involvement of GARP-dependent trafficking in acrosome formation has been demonstrated in mice (Paiardi et al., 2011). The fruit fly spermatogenesis model provides a unique opportunity to study the involvement of Golgi ribbon biogenesis in acrosome integrity and formation, since the acroblast is the only true ribbon-like Golgi structure in the developing *Drosophila* sperm. Generation of a Golgi-ribbon is known to require the microtubule mediated transport of Golgi elements to the perinuclear region (Wehland et al., 1983). Our finding that GARP complex function is required for the correct formation of the perinuclear Golgi, known as acroblast, raised the intriguing possibility that other known retrograde transport factors could also be important contributors of Golgi-ribbon formation. A second retrograde trafficking factor, the Fws subunit of the intra-Golgi transport specific COG complex, was therefore also investigated. Fws has previously been characterized during spermatogenesis using two EMS alleles (*fws^z-0161^* and *fws^z-1201^*). Transheterozygotes of these two alleles were shown to manifest in spermatocyte cytokinesis and spermatid elongation defects (Farkas et al., 2003). Given the strong defect in these EMS mutants during cytokinesis, it is unclear whether the spermatid elongation and associated acrosome formation defects are secondary consequences of the meiotic defect. We therefore decided to investigate the phenotype of a new P element insertion line *fws^KG02853^* (*fws^P^*).

*Fws^P^* contains a P element insertion in the first exon of the *fws* gene. This disruption of the *fws* gene results in 74% male sterility in homozygotes and 90% in hemizygotes over the *DfBSC148* deficiency. These numbers show that this mutant is possibly a hypomorphic allele of fws. The sterile homozygous *fws^P^* mutants' seminal vesicles were devoid of mature sperm (Fig. S1C). Remobilisation of the P element in *fws^P^* reverted the male sterile phenotype to recover complete fertility. The male sterile phenotype of *fws^P^* was also rescued with the wild type GFP-*fws* genomic rescue construct (Farkas et al., 2003), suggesting that the P element insertion in *fws* is indeed responsible for the male sterile phenotype of *fws^P^* (Fig. S1M-O). Importantly, in contrast to the previously reported EMS alleles, meiotic cytokinesis in *fws^P^* homozygous testes was normal (Fig. S1D,F) and proper elongation of the post-meiotic cysts was observed (Fig. S1F). Phenotypic characterization of developing spermatids showed scattered nuclei and investment cones in the *fws^P^* mutant cysts (Fig. 3C), but similarly to the *scat^1^* mutant the histone protamine transition was again found to be normal (Fig. 3F). Using the acrosomal markers Snky-GFP and Lamp1-GFP showed that the majority of the elongated spermatids do not form acrosomes in the *fws^P^* mutant (Fig. 3K; Fig. S2C); however, in contrast to the *scat^1^* mutant, in some cases we could observe an acrosomal signal with both transgenes with some of the acrosomes decorating non-scattered nuclei (Fig. 3K; Fig. S2C, arrowhead). This is in line with the fertility results suggesting that the *fws^P^* mutant has an overall milder defect than the *scat^1^* mutant, likely due to *fws^P^* being a hypomorphic rather than null-allele. Given the good correlation between the extent of the fertility and acrosome defects it seems that the most sensitive effect of *fws* disruption is on acrosome formation and nuclear elongation rather than cytokinesis (Farkas et al., 2003).

Finally to test if the primary defect in the *fws^P^* mutant, as in the *scat^1^* mutant, is in acroblast organization, the distribution of *cis*- and *trans*-Golgi markers was investigated in *fws^P^* from early stages up to cyst elongation (Fig. 4E; Fig. S2H,I). Similar to the *scat^1^* mutant, we found a defect in the perinuclear acroblast (Fig. 4E), however, in this instance it was the GM130 marker that showed a more dispersed staining while Golgin245 remained compact (Fig. 4E). This is in line with the COG complex, involved in intra-Golgi transport (Miller and Ungar, 2012), affecting more the formation of the *cis* side of the Golgi ribbon while the GARP complex, involved in retrograde transport to the late Golgi (Conibear and Stevens, 2000), affecting the *trans* side.

## DISCUSSION

**Fig. 5.**
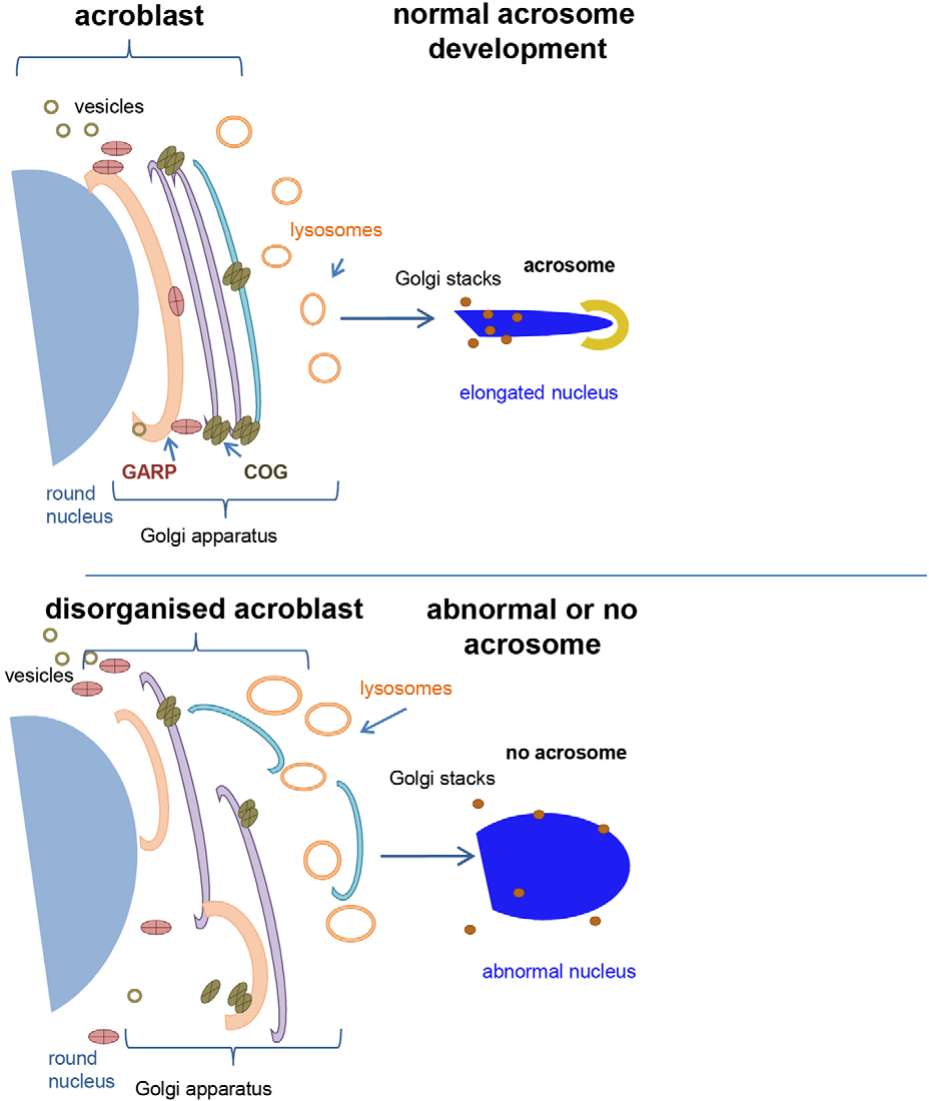
**Schematics depicting the involvement of tethering complexes in acroblast integrity.** Under wild-type conditions (top) the functions of the COG and GARP complexes in retrograde traffic are needed for the proper morphology of the Golgi ribbon known as the acroblast during spermatogenesis. This functional acroblast is then used to form the acrosome. When COG or GARP are non-functional, such as in the *fws^P^* and *scat^1^* mutants, the Golgi ribbon spreads probably due to a lack of appropriate retrograde traffic (at the *cis* side in COG mutants, or the *trans* side in GARP mutants). This results in defective acrosome formation, nuclear elongation and ultimately failed spermatogenesis.

Our work sheds light on the interplay between vesicle trafficking, Golgi structure, acrosome formation and sperm development. The primary defect during sperm development in the analysed mutants is disruption of the acroblast structure, which in turn causes defects in acrosome formation, nuclear elongation and individualization. As opposed to previously characterized Golgi trafficking mutants that have been shown to exhibit acroblast abnormalities (Farkas et al., 2003; Giansanti et al., 2006; Robinett et al., 2009), the *scat^1^* and *fws^P^* mutants exhibit no meiotic cytokinesis defects at all. The Giotto and TRAPP mutants, as well as the EMS mutagenesis alleles of *fws*, did show acroblast defects, but this could have been caused by a generic delay and consequent disruption of spermatogenesis due to defective cytokinesis. Our study is therefore the first clear demonstration that both Scat and Fws, and therefore the GARP and COG complexes, are essential for establishing correct acroblast morphology. Consequently, the lack of acrosomes in the here-described mutants is again a more direct demonstration of the need for acroblast homeostasis in order to generate the acrosome and complete nuclear elongation, since other spermatogenesis stages up to the canoe stage of nuclear elongation are normal. The acrosome has been shown to contain elements of the TGN as well as the late endosome/lysosome (Hermo et al., 2010). We find that the correct organization of the rest of the Golgi, including both the *cis* and *trans* sides, is essential for acrosome formation despite these Golgi components not being incorporated into the acrosome (Fig. 5). The mutants we describe will therefore be valuable tools in the future to study acrosome biogenesis during *Drosophila* spermatogenesis.

Several steps during post-meiotic spermatogenesis occur in parallel or close succession. These include chromatin condensation, basal body formation, acroblast formation, nuclear elongation and acrosome formation. The mutants characterized in this study allow us to place these in a hierarchy of dependence. It is clear that acroblast formation is not required for chromatin condensation, basal body formation and the initial phase of nuclear elongation. At the same time, formation of the acrosome and the elongation to needle-shaped nuclei cannot proceed even where chromatin has condensed and the basal body formed unless the acroblast is fully functional (Fig. 5). The most important function of the acroblast's intact ribbon during nuclear elongation and acrosome formation is its influence on the polarization of the cyst, which ultimately leads to normal individualization. However, molecular details of the links between acroblast formation and function and the process of individualization remain to be identified.

The acroblast is a very special form of the Golgi apparatus in *Drosophila*, since it forms a perinuclear ribbon as opposed to the scattered stacks found in other cells of the fruit fly (Kondylis and Rabouille, 2009). The two mutants analyzed in this study show normal Golgi distribution in cells where the scattered stack morphology is predominant (Fig. S2F,H). This implies that the retrograde transport routes defined by GARP and COG are not essential for the formation and maintenance of Golgi stacks in spermatocytes. While Golgi defects are common in mammalian COG mutants (Reynders et al., 2009; Ungar et al., 2002), a loss of Golgi stacks is not observed. Similarly, the ribbon of the acroblast is seriously malformed in both the *scat^1^* and the *fws^P^* mutants. This implies that ribbon formation may indeed need the retrograde transport pathways established for GARP (Bonifacino and Hierro, 2011) and COG (Miller and Ungar, 2012) in addition to the well-known contributions of microtubule-mediated anterograde transport (Wehland et al., 1983). The fact that the observed disruption in the acroblast is most prominent on either the *trans* side (for GARP) (Conibear and Stevens, 2000) or the *medial*/*cis* side (for COG) (Miller et al., 2013) is in line with the respective known destinations of the transported vesicles (Bonifacino and Hierro, 2011; Willett et al., 2013). Several candidates for the associated machinery that could act together with COG have already been flagged up by other studies, such as the golgins TMF (Schmitt-John et al., 2005) and GMAP210 (Kierszenbaum et al., 2011) that are both essential for acrosome formation in mouse testes. Yet future studies are required to address what it is that has to be delivered to the particular Golgi areas by COG- and GARP-mediated retrograde transport in order to generate specific parts of the ribbon: is it the whole vesicle that is needed, is it a very specific transport factor or factors that have to be recycled, or is it the general protein homeostasis within cisternae, maintained through recycling, that is essential for ribbon maintenance in the acroblast?

## MATERIALS AND METHODS

### Fly stocks, mutants and transgenes

Flies were crossed and maintained on standard cornmeal agar medium at 25°C. Oregon-R stock was used as wild-type control. Fertility tests were performed by crossing single males with four wild-type females. The progeny was counted in every tube and an average calculated from 30-50 males.

The following lines were obtained from the Bloomington Stock Center: *scat^1^*, *Df(2L)ED680*, *fws^P^(fws^KG02853^*), *Df(2L)ED1175*, *P(His2Av-EGFP.C2)*, *P(protamineB-eGFP)*. Flies carrying the *Snky-GFP*, *GFP-PACT*, *Lamp1-GFP, bam*-Gal4 and GFP-*fws* transgenes have been described previously (Farkas et al., 2003; Martinez-Campos et al., 2004; Wilson et al., 2006). *Snky-GFP*, *Lamp1-GFP* and GFP-*fws* transgenes were recombined onto the 2nd chromosome with *scat^1^* and *fws^P^.* Remobilization of the P element in *scat^1^* and *fws^P^* was done according to Engels et al. (1990). Revertant lines were tested for fertility and the precise excisions of the P elements were confirmed by PCR. The C-terminal *P{UASp-Scat-RFP}* construct was generated using the Gateway^®^ cloning system (Invitrogen) according to the manufacturer's instructions, using *scat* cDNA. Transgenic lines were established and the *bam*-*Gal4* testis specific driver was used to express the transgene in wild-type and *scat^1^* mutant backgrounds. The *P{tv3-Arl1-mCherry}* and *P{tv3-GFP-Lerp}* transgenic constructs were generated by amplifying the *arl1* and *lerp* cDNAs, and cloning the PCR fragments into a modified testis-vector3 (Wong et al., 2005) containing an insertion of mCherry or GFP to create a C- or N-terminal fusion protein. Transgenic flies were generated on a *w^1118^* background.

### Staining and microscopy

For immunostaining, intact or partially squashed testes from 2-4 day old wild-type and mutant flies were processed as described earlier (White-Cooper, 2004). DAPI (1 µg/ml) was used for DNA staining and Texas Red^®^-X Phalloidin (Invitrogen) was used in 1:250 dilution for actin visualization. Primary antibodies used were: rabbit anti-dGM130 (Abcam) and goat anti-dGolgin245 (1:100, gift of Sean Munro, MRC-LMB Cambridge; Riedel et al., 2016).

Alexa Fluor 488 and Alexa Fluor 594 conjugated anti-rabbit secondary antibodies were from Invitrogen. The samples were mounted in Fluoromount (Southern Biotech) and imaging was done with an Olympus BX51 fluorescent microscope or an Olympus FV 1000 confocal microscope. Nuclear length was measured by ImageJ (NIH), statistical significance of differences determined using a one-way ANOVA on ranks with a Tukey post-hoc test.

### Antibody generation and western blotting

Polyclonal antibody was raised in guinea pigs immunised using purified His-tagged fusion protein containing the N-terminal 200 residues of Scat expressed from the pET28b vector (Novagen). For immunoblotting analysis adult testes from 40 individual males of each genotype were homogenised in 100 µl of lysis buffer (10 mM Tris-HCl pH 7.5, 150 mM NaCl, 0.5 mM EDTA, 0.5% NP40, 1 mM PMSF, 1× protease inhibitor cocktail) at 4°C. Samples were separated on 8% SDS polyacrylamide gels (Bio-Rad) and transferred to PVDF membrane for immunoblotting. Blocking and antibody incubations were in Tris-buffered saline (Sigma-Aldrich) with 0.05% Tween-20 (TBST) containing 4% nonfat dry milk. Primary antibodies were anti-Scat diluted 1:2000 and anti-tubulin (Abcam). HRP-linked secondary antibodies (Millipore) were used at 1:5000. After incubation with the antibodies, blots were washed in TBST and imaged on X-ray film using an ECL detection kit (GE Healthcare).
